# A Systematic Review and Illustrative Case Presentation of Low-Grade Myofibroblastic Sarcoma (LGMS) of the Extremities

**DOI:** 10.3390/jcm12227027

**Published:** 2023-11-10

**Authors:** Astrid Schenker, Ewgenija Gutjahr, Burkhard Lehner, Gunhild Mechtersheimer, Eva Wardelmann, Rosa Klotz, Eva Kalkum, Marcus Schiltenwolf, Leila Harhaus, Tobias Renkawitz, Benjamin Panzram

**Affiliations:** 1 Department for Orthopaedics, University of Heidelberg, Schlierbacher Landstraße 200a, 69118 Heidelberg, Germany; burkhard.lehner@med.uni-heidelberg.de (B.L.); marcus.schiltenwolf@med.uni-heidelberg.de (M.S.); leila.harhaus-waehner@med.uni-heidelberg.de (L.H.); tobias.renkawitz@med.uni-heidelberg.de (T.R.); 2 Department of Pathology, University of Heidelberg, 69120 Heidelberg, Germany; ewgenija.gutjahr@med.uni-heidelberg.de (E.G.); gunhild.mechtersheimer@med.uni-heidelberg.de (G.M.); 3 Gerhard Domagk Institute of Pathology, University Hospital Muenster, Albert-Schweitzer-Campus 1, Building D17, 48149 Muenster, Germany; eva.wardelmann@ukmuenster.de; 4 Study Center of the German Society of Surgery (SDGC), University of Heidelberg, Im Neuenheimer Feld 130.3, 69120 Heidelberg, Germany; rosa.klotz@med.uni-heidelberg.de (R.K.); eva.kalkum@med.uni-heidelberg.de (E.K.); 5 Department of General, Visceral and Transplantation Surgery, Heidelberg University Hospital, 69118 Heidelberg, Germany

**Keywords:** LGMS, soft tissue sarcoma, myofibrosarcoma, thumb reconstruction surgery, Holevich’s flap

## Abstract

Introduction: Low-grade myofibroblastic sarcoma (LGMS) is a rare tumor entity which occurs in the subcutaneous and deep soft tissues; it is less common in the bone with a predilection for the extremities and the head and neck region. As confirming the diagnosis is difficult and treatment strategies are not standardized, we aimed to identify patient and tumor characteristics, and to summarize treatment strategies and their clinical outcomes to guide surgeons. Methods: Included were full articles reporting patients with histology of LGMS in the extremities, excluding tumors of the trunk. All patients underwent surgery but with different extend, from marginal to wide resection. Included studies should inform about local recurrence, metastasis, or evidence of disease, depending on the surgical treatment. We conducted a structured search using MEDLINE (via PubMed), Web of Science, EMBASE and Cochrane Central Register of Controlled Trials (CENTRAL) to identify studies on low-grade myofibroblastic sarcoma of the extremities. Study designs like randomized controlled trials, systematic reviews, prospective trials, retrospective studies, and case reports were included. Prospective studies and comparative studies were not available at all. Therefore, meta-analysis was not possible and statistical analysis was purely descriptive. Results: Of the 789 studies identified from our initial search, 17 studies including 59 cases reported LGMS of the extremities with the surgical treatment and clinical outcome and were therefore analyzed. In addition, we present the rare case and surgical management of a 28-year-old male patient with residual LGMS of the thumb after an initial incomplete resection. The current literature suggests that a wide excision with R0 margins should be considered the standard treatment for LGMS. In cases where surgery leads to significant functional impairment, individual options like free tissue transfer from a donor site have to be considered. Therefore, we also present an illustrative case. For all selected case series and case reports, a high risk of confounding, selection bias, information bias, and reporting bias must be anticipated. Nevertheless, this systematic review provides a comprehensive overview on surgical treatment and clinical outcomes in LGMS surgery of the extremities.

## 1. Introduction

Low-grade myofibroblastic sarcoma (LGMS) is an infrequent, well differentiated, and atypical neoplasia characterized by a growth pattern reminiscent of fibromatosis. Although a sarcoma displaying ultrastructural myofibroblastic features was initially described by Vasudev and Harris in 1978 [1,2], its definitive identification as a distinct neoplasm awaited Mentzel et al.’s comprehensive characterization in 1998 [3]. Subsequently, in 2002, the World Health Organization incorporated it into the International Classification of Diseases for Oncology (ICD-O) [1,2]. LGMS exhibits a broad anatomical distribution, with a predilection for the extremities and the head and neck region, lacking a specific age predisposition [3,4,5,6,7]. The primary tumor mass is frequently located within subcutaneous and deep soft tissues, displaying an aggressive biological behavior with a tendency to cause local recurrence, but rarely metastases [8]. Chan et al. (2016) reported, in his population-based study, a 5-year overall survival rate of 71.6% among patients diagnosed with LGMS in the United States [4]. While the precise incidence of the tumor remains unknown, it is presumed to be more prevalent than previously anticipated due to the diagnostic challenges it presents.

Imaging diagnostics for LGMS typically include X-ray, MRI (with or without contrast materials), and CT scans. Imaging features of the non-specific soft tissue mass can vary depending on the tumor’s location. In addition to calcifications or ossifications, lesions with bone involvement may appear as osteolytic and destructive masses [9]. On MRI, the mass is typically hypo- to iso-intense to muscle in T1 images, while in T2 images it exhibits heterogeneous high signal intensity [9,10]. Most cases of LGMS form a firm mass with a pale, fibrous cut surface and usually ill-defined margins [1]. Its highly heterogeneous histological aspect makes it difficult to diagnose and to differentiate LGMS from other benign or malignant lesions [11,12]. Histologically, most low-grade myofibroblastic sarcomas are characterized by a diffusely infiltrative growth pattern without a sharp border to the surrounding tissue, often interspersing between pre-existing skeletal muscle fibers. The cell density of the tumor varies highly, from the rarest hypocellular, scar-like cases with a prominent collagenous (partly hyalinized) matrix, to more common cases with variably compactly packed atypical spindle cells showing a storiform growth pattern or an arrangement in short fascicles with variable myxoid and collagenized regions [1]. The myofibroblast-like neoplastic cells have ill-defined pale, eosinophilic cytoplasm and nuclei that are, depending on their mitotic activity, either elongated and wavy with evenly distributed chromatin, or rounded and plumper with indentations and small nucleoli [13]. The obligatory diagnostic criterion is the proof of singular mitotic figures, as well as of cellular atypia. Intratumorally, several blood vessels with thin walls are included; an inflammatory superposition is only focally observed in singular cases in the form of small aggregates of plasma cells, eosinophils, and lymphocytes [14]. Dystrophic calcifications and osseous metaplasia are uncommon but can occasionally be observed [15,16,17]. Necrosis is also untypical. If looking at the results of immunohistochemical staining and genetic analyses, the bandwidth of heterogeneity of LGMS will additionally emphasize the difficulty of diagnosing this tumor entity, as mentioned above. According to Mentzel et al. [13], LGMS shows a variable expression of smooth muscle actin (SMA) and desmin, and can focally be positive for calponin as well as for CD34. The stainings for epithelial markers β-catenin, protein S-100, and h-caldesmon are usually negative. Genetic aberrations have been described in only a few cases, thus lacking any specificity [14]. Nevertheless, the use of electron micrographic analysis in singular cases during the routine diagnostics could definitely help to determine the LGMS diagnosis. Discontinuous basal lamina and thin filaments with focal densities and subplasmalemmal attachment plaques, as well as micropinocytic vesicles, are absolutely specific for LGMS. However, the limited availability of this analysis and the high cost of the method strongly limit the potential of electron microscopy [13].

Due to all the limiting factors in the histopathological diagnostic routine described above and the challenge in distinguishing between tumor subentities with similar benign, intermediate, and malignant potential, the distinct clinical behavior of most soft tissue and bone sarcomas serves as a crucial determinant for devising treatment protocols [18,19]. In the context of LGMS, which shares these diagnostic complexities, establishing definitive treatment criteria remains an ongoing endeavor. The objective of this study is to conduct a systematic review encompassing all pertinent instances of LGMS affecting the extremities. This comprehensive analysis seeks to shed further light on this rare neoplastic entity to assess the most effective treatment approach for LGMS. To illustrate this, we present an illustrative case of LGMS occurring in the thumb, wherein the challenge of achieving a wide resection underscores the necessity of a tailored treatment strategy adjustment. To the best of our knowledge this is the first systematic review of LGMS of the extremities.

## 2. Materials and Methods

In this systematic review, we identified studies that elucidate and evaluate the surgical management and clinical outcomes associated with LGMS affecting the extremities for patients who underwent surgery, varying from marginal to wide resection. The included studies inform about local recurrence, metastasis, or evidence of disease depending on the surgical treatment. Study designs like randomized controlled trials, systematic reviews, prospective trials, retrospective studies, and case reports were all accepted for inclusion. This review complies with the recommendations of the Cochrane Handbook for Systematic Reviews of Interventions [20], and is reported in line with the PRISMA guidelines [21]. There was no external source of funding. Two of the authors independently screened the title and abstracts of all retrieved references. In cases where clarification was needed, a consensus was reached through discussion.

### 2.1. Literature Search

**Figure 1:**
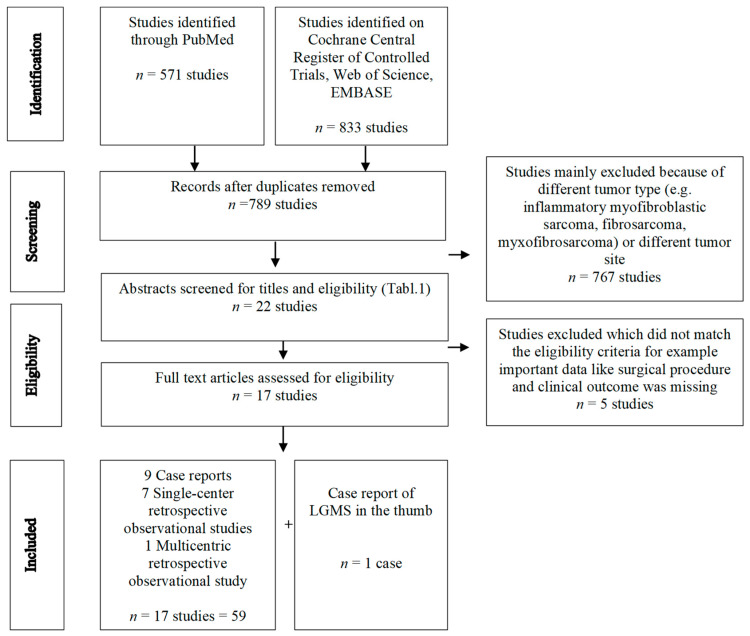
PRISMA flow diagram.

We systematically screened MEDLINE (via PubMed), Web of Science, EMBASE, and Cochrane Central Register of Controlled Trials (CENTRAL) from 1946 to 2023 to identify studies on LGMS of the extremities. The search strategy contained the subject heading low-grade myofibroblastic sarcoma. MESH terms included fibrosarcoma/surgery and extremities. The full search strategy is displayed in Table 1. Included were abstracts and full articles reporting patients with histology of LGMS in the extremities, excluding tumors of the trunk. A PRISMA flow diagram outlines each stage of the review (Figure 1). In addition, we searched the references of the included articles to find relevant studies. Non-English literature and inaccessible literature were excluded.

### 2.2. Data Extraction and Analysis

After removing duplicates we used the eligibility criteria (Table 2) for abstracts and full texts to find relevant studies. Afterward, full-text articles for each of the selected abstracts were analyzed. Data extraction included patient characteristics (mean age, sex distribution, and tumor size), treatment regimen (type of surgery, neoadjuvant/adjuvant therapy), and efficacy endpoints (local recurrence, metastasis, no sign of recurrence, dead of disease, and alive with evidence of disease) if available in the publication (Table 3a,b).

### 2.3. Quality Assessment and Evaluation of the Risk of Biases

Table 4 displays the bias risk that has been evaluated with the help of the Critical Appraisal Checklist for Case Reports developed by Moola et al. [33]. Most of the included studies were case reports. We also analyzed the cases mentioned in the clinicopathological study or the small retrospective studies, as well as those mentioned in the case series according to the Checklist for Case Reports, to guarantee equality in study analysis. If a case met at least 4 of the 8 appraisal criteria, it was considered to be of acceptable quality to be included in the systematic review.

## 3. Results

A total of 1404 studies was obtained from the systematic literature search. After removing duplicates and using the eligibility criteria (Table 2) for abstracts and full texts, we identified 17 studies, including 59 cases of operatively treated low-grade myofibroblastic sarcoma of the extremities. Table 3a summarizes the cases of LGMS located in the superficial and deep soft tissue while Table 3b shows cases of LGMS in the bone. The detailed selection process is illustrated in the PRISMA Flowchart (Figure 1).

### 3.1. Study Characteristics

Existing literature predominantly comprises case series, case reports, a limited number of retrospective observational studies, analytical investigations utilizing the Surveillance, Epidemiology, and End Results (SEER) database, and a retrospective multicenter study conducted by the Japanese Musculoskeletal Oncology Group. 

### 3.2. Patient and Tumor Characteristics

The mean age of the patients was 43.6 years, with a range spanning from 6 to 86 years. A balanced male-to-female ratio of 1:1 was observed, as indicated in the available studies. Detailed patient and tumor characteristics are summarized in Table 3a,b. The most prevalent clinical presentation involved the emergence of a painless, progressively enlarging mass. Of all cases, 29 tumors (47%) originated in the upper extremities, encompassing the shoulder girdle, while 34 cases (54%) were localized to the lower extremities and groin. A singular instance of multicentric LGMS involving both upper and lower extremities was recorded (case 16). There were 44 cases located within soft tissues (Table 2), with 15 cases situated within bones (Table 2), demonstrating a particular predilection for the femur. A limited number of cases involving LGMS in the hand were documented, specifically cases 13, 48, and 58. Tumor size ranged from less than 1 cm up to 20 cm, with multiple nodules in some cases.

### 3.3. Imaging Features

**Figure 2:**
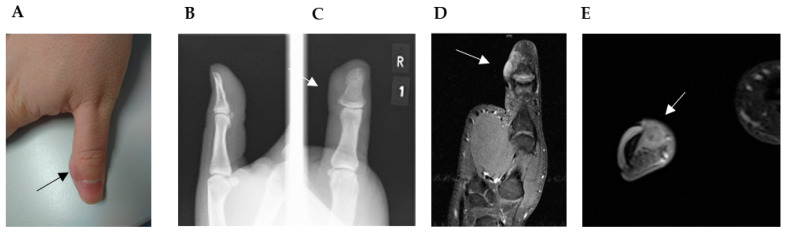
(**A**) Straight to ulnar view of the thumb with residual soft tissue mass, approximately 2 × 1.5 cm in diameter and located on the ulnar side of the distal phalanx of the right thumb (→). (**B**) Lateral and (**C**) anteroposterior plain radiographs revealed no bone erosion close to the soft tissue mass (→). (**D**,**E**) Magnetic resonance imaging (MRI) with contrast material: (**D**) coronal, (**E**) transversal view of the predominantly hyperintense on T2-weighted images. (**D**) Images revealed an irregular mass located directly adjacent to the cortical bone without signs of invasion or infiltration and without alteration of the intramedullary bone signal (→).

Concerning extremities, radiologic findings showed, in some cases, bone-destructive lytic lesions and calcification within the tumor [9,22,27,29]. Although the margins were comparatively clear, cortex destruction with soft tissue extensions was common in those cases. Periostal reaction was not observed. Saito et al. presented a rare case of LGMS of the bone showing a honeycombed lucent lesion on plain radiography of the right distal femur [32]. Other cases, like ours, present only soft tissue mass without any bone reaction (Figure 2B–E). On MRI, LGMS presents as a soft tissue mass heterogeneously hyperintense in the T2-weighted image and hypo- or iso-intense in the T1-weighted image (WI) (Figure 2D–E) [7,10,31]. In some cases edema occurred in the surrounding tissue [7].

### 3.4. Treatment Strategy

All 59 documented cases underwent surgical intervention as part of their treatment regimen. The spectrum of treatment modalities encompassed a range from local excision to wide resection, though without a clearly defined safety margin. Among these cases, wide resection was performed in 20 cases (34%), with an additional 5 cases (8%) subsequently undergoing radiotherapy, and 2 cases (3%) receiving adjuvant chemotherapy. In two cases, prosthetic implantation was employed following wide resection of the tumor. Marginal or local excision, including intralesional resection, was noted in 15 cases (25%), with 2 cases supplemented by adjuvant radiotherapy. Notably, three cases culminated in amputation (cases 7, 45, and 48). In 12 instances (20%), the specific surgical procedure was not explicitly detailed. In total, 12 patients (20%) received adjunctive therapy, either radiotherapy or chemotherapy, subsequent to their surgical intervention.

### 3.5. Clinical Outcome

Follow-up information was obtained from 47 patients, with a mean follow-up time of 51 months (range 6–192 months). In total, 13 (22%) suffered from local recurrence, while 1 patient even had eight recurrences in 8 years (case 58). In one case, local recurrence occurred after wide resection and chemotherapy, and was treated by radiotherapy (case 7). One case mentioned transformation of IMT into LGMS after three recurrences (case 19). Another local recurrence after 70 months led to amputation of the leg. In case 45 of this study, another amputation was performed after local recurrence in the tibia. Five cases (10%) showed distant metastasis in the follow-up time from 8–56 months, while one case with metastasis was identified at time of diagnosis (case 54). Three of them occurred in the lung (cases 54, 55, and 56) and one in the bones (case 57). One case described a tumor-related death with cardiac metastasis (case 15).

### 3.6. Case Presentation and Clinicopathological Features

A 28-year-old male patient presented at our clinic with a residual thumb tumor. Initially, he noticed a gradually growing mass on his thumb in September 2019 and subsequently sought medical attention in October at an external clinic due to the presence of a tumor mass on the distal phalanx of the thumb. There was no history of prior skin lesions or trauma. An X-ray examination of the thumb did not reveal any evidence of bone infiltration, and an initial MRI was not conducted. The tumor was excised without prior biopsy. In December 2019, two months post-surgery, the patient presented at our hospital for the first time, reporting recurrent swelling at the surgical site. A comprehensive examination indicated that the patient was in good general health and denied any tobacco or alcohol usage. The thumb displayed an elastic nodular tumor mass on the distal ulnar aspect of the phalanx (Figure 2A). The physical assessment detected no palpable lymph nodes in the axillary, supraclavicular, or head and neck regions. X-ray imaging demonstrated no evident skeletal alterations (Figure 2B,C). MRI findings revealed two closely situated soft tissue nodules, measuring 1.8 × 0.9 cm in diameter, with the proximal nodule displaying homogeneous enhancement. These MRI characteristics raised significant suspicion of a recurrent or residual tumor (Figure 2D,E) of unknown malignancy. Further assessment through chest computed tomography exhibited no indications of metastatic involvement. Hematologic parameters and biochemical markers fell within normal ranges.

**Figure 3:**
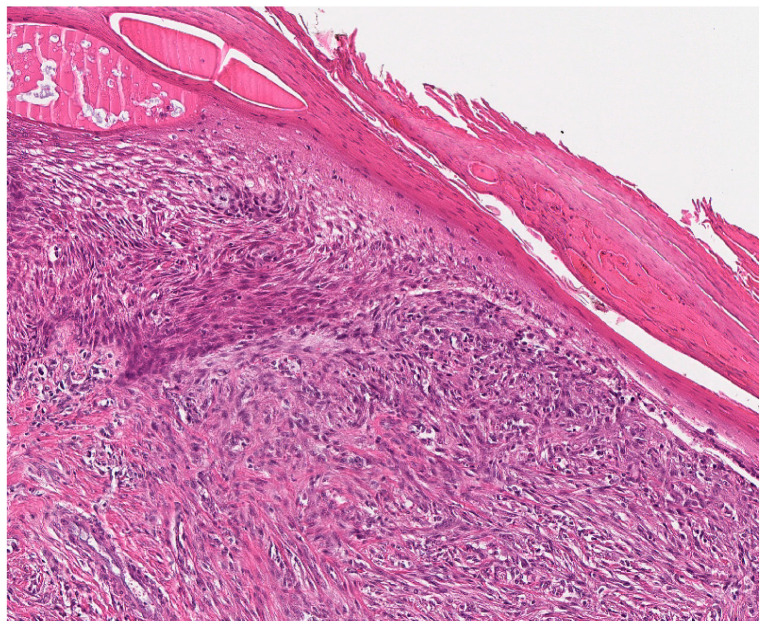
Histological aspects of low-grade myofibroblastic sarcoma. LGMS, characterized by a high cell density in this case, is composed of fusiform tumor cells with spindle-shaped, monomorphic, vesicular nuclei with small nucleoli and ill-defined, pale eosinophilic cytoplasm.

Our presented case (case 33) showed an approx. 0.9 × 0.8 × 0.6 cm measuring recurrent tumor with a yellowish-beige cut surface and diffuse borders to the surrounding soft tissue. It was identified in the deeper dermis and subcutis of the finger partial amputate within the macroscopic evaluation at the Institute of Pathology, University Hospital Heidelberg. Histologically, the recurrent tumor showed a diffusely invasive growth pattern with perineural tumor growth and enclosure of intradermal Vater-Pacini bodies, as well as a focal relation to epidermis. It consisted of atypical, compactly packed spindle cells with an arrangement in shorter fascicles, and was embedded in a hyalinized collagenous matrix (Figure 3). The monomorphic tumor cells had elongated nuclei with singular prominent nucleoli and minimal pleomorphism, and were surrounded by a pale, eosinophil cytoplasm. No tumor necrosis, vascular or lymphatic invasion were observed. Intratumorally, thin-walled blood vessels were interspersed. The conventional light microscopic aspect of the spindle cell neoplasia of the finger partial amputate was compatible with a completely resected recurrent low-grade myofibroblastic sarcoma. No additional staining was performed. The diagnosis of LGMS (FNCLCC Grade 2 (2 + 2 + 1)) became the favorite exclusion diagnosis and was primarily supported by the conventional histomorphological aspect of the neoplasia.

The primary tumor (resected and diagnosed at the Municipal Hospital of Karlsruhe and the University Hospital of Münster), located in the deeper dermis and subcutis, showed a similar histological aspect, however, including a central tumor necrosis and several mitoses up to highly pleomorphic nuclei (ca. 5 mitoses/HPF). Due to the combined nuclear pleomorphism and infiltrative growth pattern, the differential diagnoses of fibromatosis and nodular fasciitis could be excluded. In the context of confirming the local recurrence, the fact that the surgical margins of the primary tumor resection were not free of tumor is to be emphasized. Extensive immunohistochemical staining of the primary tumor was performed at the Department of Pathology, University Hospital of Muenster. The neoplastic cell population showed a homogenous expression of the myogenic markers smooth muscle actin (SMA) and desmin (additionally contradicting the diagnosis of fibromatosis), accompanied by a weaker immunoreactivity of CD10, as well as vascular markers ERG (strong), CD34 (microfocal, weak), and D2-40. The INI1 expression was preserved. The immunohistochemical staining was negative for several other markers (Table 5). The proliferation activity (Ki-67-index of approx. 15–20%) was relatively low. Thus, the moderate neoplastic expression of myogenic markers, as well as the lack of the track tram growth pattern, the lower eosinophil and more tender tumor cells in this case, were used to delimit the differential diagnosis of leiomyosarcoma. The immunonegativity against ALK-1 helped to exclude the diagnosis of an inflammatory myofibroblastic tumor, as well as the absence of lymphoplasmatic aggregates scattered among tumor cells and the cellular atypia of the resected tumor. Whereas a weak reactivity of the tumor against *TFE3* was observed immunohistochemically, no translocation of the *TFE3* gene locus was identified by fluorescence in situ hybridization (FISH) using dual color break apart gene probes. Thus, the diagnosis of the alveolar soft part sarcoma was cancelled. Finally, the FISH investigating the chromosome 22q13 could not verify the presence of a *PDGFB* translocation. In the synopsis of this result, as well as considering the proof of the fusion *COL1A2::GNS* (exon 6/exon 4) by RNA sequencing, which has not been described in literature so far, the diagnosis of dermatofibrosarcoma protuberans seemed improbable, and was supported only by microfocal weak immunoreactivity against CD34 and the lack of plaque-like neuroid neoplastic areas. Despite a weak diffuse positivity for CD34 (in ca. 10% of tumor cells), negativity for STAT6, several nuclear atypia and the lack of patternless haphazard arrangement of the neoplastic cells, and the lack of *NAB2::STAT6* fusion transcripts, the diagnosis of a solitary fibrous tumor (SFT) could also not be confirmed.

**Figure 4:**
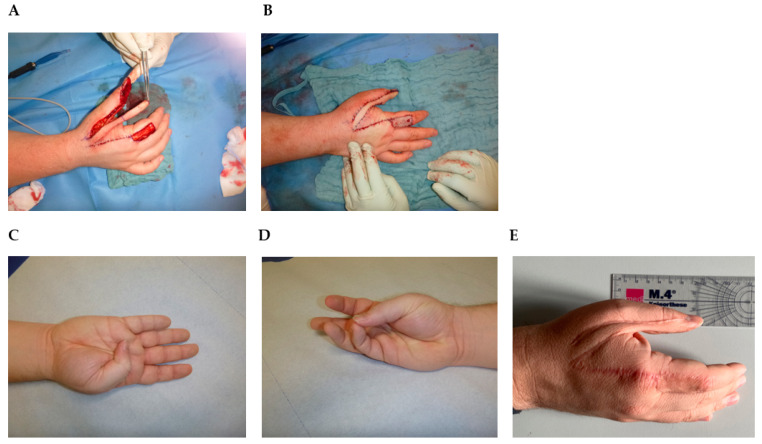
Holevich’s flap. (**A**) Flap elevation after tumor resection. (**B**) Reconstruction with the flap. The donor area is covered by an antecubital full-thickness skin graft. (**C**) Thumb flexion 12 weeks after operation. (**D**) Opposition to the little finger 12 weeks after operation. (**E**) Ulnar view 38 months after operation.

Considering the intermediate-grade biological behavior of the tumor, with early recurrent growth after an initially incomplete resection, the young age of the patient, and the importance of the thumb for the hand function, a two-stage surgical intervention was indicated in our case. In the first step, a wide excision was performed to obtain clear resection margins. Soft tissue was resected, including the ulnar half of the bony distal phalanx (Figure 4A). Temporarily, the tissue was covered with synthetic skin replacement. The histopathologic analysis confirmed the diagnosis of a low-grade myofibroblastic sarcoma. Soft tissue resection margins were negative without tumor infiltration of the bone. So we performed reconstruction of the defect with a neurovascular Holevich’s flap (Figure 4A,B) [34]. The island flap was prepared from the dorsum of the index finger, with a proximally based skin pedicle [35,36]. The flap included the first dorsal metacarpal artery (DMCA) with concomitant veins and the terminal branches of the superficial radial nerve, which provided stable soft tissue cover to the bone, as well as the preservation of fingertip sensation. The donor area was covered by an antecubital full-thickness skin graft. The wound showed primary wound healing. The flap survival was total with good flap sensibility and donor site sensibility. The thumb had good function, with slightly reduced flexion of the IP joint (Figure 4C), and opposition to the other four fingers was possible (Figure 4D). The patient showed good grip strength and a stable precision grip. MSTS-Score, which measures pain, function, emotional acceptance, hand positioning, manual dexterity, and lifting ability, showed a good result of 95%. Tumor control after 38 months showed good wound healing, and no local recurrence or distant metastasis on local MRI and chest X-ray (Figure 4E).

## 4. Discussion

Low-grade myofibroblastic sarcoma (LGMS) represents a particularly uncommon and often underestimated tumor type that can manifest within the extremities. The reported incidence of this tumor is thought to surpass previous estimations due to the inherent challenges associated with its precise diagnosis. Given the limited number of small-scale clinical studies and individual case reports in existence, it becomes imperative to convey the primary insights concerning diagnosis, surgical management, and outcomes through a comprehensive review of the available collective experiences. This endeavor serves a dual purpose: enhancing clinical practices within the context of rare diseases, and furnishing a more comprehensive informational foundation for clinicians to make informed treatment decisions [3,4,14,33].

In the current study, we have systematically extracted and amalgamated the principal demographic and clinical attributes characterizing LGMS occurrences in the extremities from the extant body of literature. Additionally, we share our own encounter with an exceedingly rare instance of LGMS affecting the thumb. To the best of our knowledge, no publication has undertaken the task of aggregating reported instances of LGMS within the extremities in a quantitatively structured manner.

Different literature reviews describe the location distribution as follows: most cases occur in the head and neck region, followed by the trunk and extremities [7], but LGMS can occur in almost every region of the body [29,37,38,39,40,41,42]. We have identified and analyzed 59 cases in the extremities including the groin and shoulder girdle. As in other locations and previous publications, we found no distinct age preference, even though most of the patients were <60 years old (63%), and roughly equal gender incidence. A multivariate analysis from Chan et al. [4] showed that older patient age was significantly associated with worse survival (*p* < 0.05). Moreover, Chan et al. noticed a significantly different tumor size comparing head and neck LGMS and non-head and neck LGMS, the latter having a significantly greater number of cases with tumor size > 4 cm [4]. Non-head and neck tumors were present in the abdomen and pelvis or extremities, allowing the tumor to grow silently before symptoms presented [43]. Kito et al. [28] showed an association between tumor size and local relapse in his multicenter study investigating different tumor localizations. Tumors in the head and neck region are more easily visible, leading to earlier diagnosis and therapy. Concerning only cases of LGMS in the extremities, we also see a tendency for local recurrence for tumor size > 4 cm (Table 3), with the exception of the hand, where a smaller tumor mass can lead to a local recurrence.

Establishing uniform diagnostic criteria for this rare tumor, using both MRI and histopathological evaluation, has remained a challenge. However, the inclusion of preoperative MRI is crucial in the evaluation of soft tissue sarcoma [44]. It can help to prevent unnecessary aggressive surgical procedures and to distinguish LGMS from benign lesions, even if the MRI findings exhibit non-specific features [30]. In the presented studies, where LGMS occurred in bones, osteolytic lesions were observed, which is more likely to indicate a malignant tumor [9,27,29,30,32]. Typically, there were no periosteal reactions but calcifications could be detected within the tumor [9,27,29]. Concerning synovial sarcoma, the absence of calcification is associated with reduced disease-free survival [44]. To what extent this applies to LGMS cannot be seen from the literature or the analyzed data. MRI revealed that a T1-weighted image (WI) signal was mostly an equal signal, whereas LGMS presents as a soft tissue mass heterogeneously hyperintense in T2-weighted images. Signal heterogeneity is associated with the worst prognosis in all types of soft tissue sarcoma [44]. Morii et al. [45] and Niu et al. [46] reported the usefulness of 18F-Fluorodeoxyglucose-positron emission tomography (FDG-PET)/computed tomography (CT) for diagnosing LGMS. As far as we know, this method was not described for LGMS of the extremities. They suggested that the high capacity of glucose utilization is a possible reflection of LGMS.

The complexity of arriving at a definitive diagnosis necessitates a combined approach involving histopathological and immunohistochemical analyses. Strict determination of the grade of nuclear pleomorphism in each tumor is difficult because of the wide range of histologic features [10,23].

During the diagnostic process, consideration should be given to incisional biopsy as it facilitates an accurate diagnosis and justifies the invasiveness associated with subsequent surgical interventions [15]. For lesions of diminutive size, the decision to perform an excisional biopsy should be approached with caution. Ideally, such a procedure should only be undertaken if the prospect of complete resection with negative margins is deemed feasible, thereby mitigating the risk of local recurrence [10,31]. This recommendation draws from experiences in other tumor types and is further reinforced by the observations presented in case reports. It is important to acknowledge that the available sample sizes in these reports might not be sufficient for a comprehensive assessment.

The most common treatment for LGMS in the studies described here was wide excision. Most studies emphasize excising the tumor with an adequately wide margin [10,26]. But when it comes to the safety distance, the data situation is inconsistent. Kim et al. suggests a routine resection margin of 3 cm, adjusted according to the location of the tumor or the surrounding structures [27]. Nakashima et al. reports in his case that MRI after preoperative radiotherapy showed the reduction of the tumor size (reduction rate 34%), and the extent of high signal intensity around the tumor was reduced on fat suppression T2-weighted images [24]. They performed wide resection of the tissue surrounding the tumor with a 3 cm margin from the edema area, although almost the full length of the patellar tendon had to be resected. Data for assessing an appropriate margin in order to predict the risk of local recurrence are inconsistent and still debated [47]. A recent consensus practice guideline remarked that “no available evidence-based data addressed how to adequately assess margins” [48].

In this study, we found a local relapse of 22% concerning LGMS of the extremities. Concerning LGMS of different locations, we find a range of recurrence from 13.3–44.4% in the literature [3,16,22,27]. We found 31% of the patients with local recurrence were treated with local/marginal excision and 23% with wide excision, whereas information about exact surgical procedure is missing for 6 patients. To date, there are still case reports (case 59) emphasizing marginal excision as adequate treatment for this tumor entity [7]. A short follow-up time minimizes the clinical relevance of this observation. The literature proposed that a recurrent tumor can easily lead to distant metastasis and, theoretically, can escalate to a higher-grade (case 19) [49]. In their histopathological investigation, Mentzel et al. [3] noted that a recurrent instance exhibited no elevated cellularity, nuclear atypia, or heightened proliferative activity when juxtaposed with the primary lesion. This observation lends support to the notion that local recurrence primarily stems from surgical procedures and their associated margins. Conversely, their study identified augmented cellularity and an increased mitotic rate in the context of metastatic lesions. Our own examination revealed the presence of distant metastases in 10% of the documented cases of LGMS within the extremities. It is worth noting that previous reports have depicted a range of 0% to 9.1% for the incidence of distant metastasis in similar cases [14,30,32]. For intermediate-grade soft tissue tumors displaying a propensity for local recurrence rather than distant metastasis, we propose a follow-up regimen involving regular screenings. Our recommendation involves follow-up appointments every 4 months during the first-year post-surgery, followed by intervals of 6 months until the fourth year, and subsequently transitioning to annual check-ups until the 10th year after the surgical procedure. This monitoring plan should encompass local MRI and chest X-ray assessments.

While some practitioners have employed radiotherapy or chemotherapy as adjunctive treatments [35], a comprehensive study by Xu et al. [36] and other corroborative research have collectively indicated the absence of substantial evidence supporting pre- or postoperative radiotherapy or chemotherapy for LGMS patients, especially when negative surgical margins have been achieved [34,37,38]. The consideration of individualized adjuvant or neoadjuvant therapeutic avenues should be contingent on interdisciplinary discussions within a tumor board framework, especially in instances where complete resection of the tumor mass is not feasible. Cases observed in diverse anatomical locations have demonstrated favorable outcomes with a short follow-up period subsequent to partial resection and radiotherapy [28,39]. Furthermore, Xu et al. [36] documented instances where patients underwent radiotherapy in the absence of surgical intervention, although comprehensive clinical outcome details were not provided. The prognosis of this tumor is likely influenced by multifaceted factors such as tumor size, stage, location, and the overall health status of the individual, even if more comprehensive studies addressing these aspects are currently lacking.

Distinguishing LGMS of the hand, particularly the thumb, from other tumorous lesions within the extremities, is imperative due to the anatomical proximity to critical structures, resulting in the need for meticulous resection to achieve adequate margins, often requiring subsequent tissue reconstruction. In the context of hand LGMS, only three case reports have been documented (case 13, 30, and 48). Initially, the painless nature of the tumor can lead to misdiagnosis as a benign lesion, subsequently resulting in incomplete resection and residual tumor, thus contributing to local recurrence, as illustrated in our presented case. The rapid occurrence of local recurrence within two months of the initial resection indicates the potential for an inadequate initial procedure. In our approach, we employed a two-stage surgical intervention to ensure negative margins. The presented Holevich’s neuro vascular flap is a common and reliable choice at this anatomical region to simultaneously provide defect coverage and preserve local sensibility without compromising surgical oncologic principles (Figure 4). Most important for flap survival is a careful pedicle preparation, elevation, and prevention of pedicle strangulation. As presented in the study of San Miguel et al., amputation is also a feasible surgical option but leads to functional loss and aesthetic impairment [30]. Whenever possible in younger patients, limb salvage should be the first option. But data are sparse concerning clinical outcomes and patient satisfaction. In 2011, Puhaindran et al. investigated 23 patients in a retrospective study indicating that clinical outcomes regarding surgical management for malignant tumors of the thumb lead to similar results, comparing patients with thumb amputation at the interphalangeal joint and thumb-sparing wide excision with reconstruction [50]. Nevertheless, younger patients especially decline amputation. It is worth noting that sarcoma surgery, accompanied by subsequent flap reconstruction, has demonstrated a high survival rate and a low recurrence rate across various tumor locations [37].

Some important limitations should be noted. Firstly, LGMS is a rare tumor entity and evidence on therapy and prognosis of the disease is sparse. Except for one multicenter retrospective study, only small retrospective studies, case reports and case series could be included in this systematic review. Prospective studies and comparative studies are not available at all. Therefore, meta-analysis was not possible and statistical analysis was purely descriptive. For quality assessment, we used the critical evaluation checklist by Moola et al. [33] for case reports and incorporated it for the evaluation of risk of bias in the included studies. For each case mentioned in Table 3a,b we answered the suggested 8 questions. Most cases met 5 of the 8 appraisal criteria and, therefore, were considered to be of acceptable quality to be included in the systematic review. Only the cases reported by Wang et al. did not specify the exact kind of operation and, therefore, met only 4 of the 8 appraisal criteria [9] (Table 4). Nevertheless, we decided to include these studies because of additional information. However, for all case series and case reports a high risk of confounding, selection bias, information bias, and reporting bias must be anticipated. Another limitation of this review is the different or missing follow-up periods, in some cases ranging from six months up to eight years (Table 3a,b).

## 5. Conclusions

LGMS is a rare neoplasm seldomly located in the extremities. Imaging and clinical appearance frequently lead to misdiagnoses as a benign lesion, with consequent insufficient resection and local recurrence but rarely metastasis. Therefore, initial complete resection with negative margins should be the primary oncological goal. Concerning tumors of the hand and limb, function preserving surgery is desirable. Neurovascular flap reconstruction can be used to sufficiently cover the defect and preserve sensibility.

Prospective studies are necessary to provide more information about standardized surgical therapy.

## Figures and Tables

**Table 1:** Search strategy (7 March 2023).

Search strategy Pubmed
“myofibroblastic sarcoma”[tiab] OR “myofibroblastic sarcomas”[tiab] OR myofibrosarcoma*[tiab] OR “Fibrosarcoma/surgery”[Mesh] AND “Extremities”[Mesh] NOT (animals [mh] NOT humans [mh]) 571 hits
Search strategy Embase
‘myofibroblastic sarcoma’ OR ‘myofibroblastic sarcomas’ OR myofibrosarcoma* OR ‘fibrosarcoma’/’surgery’/exp AND ‘extremities’/exp 411 hits
Search strategy Central (Cochrane Central Register of Controlled Trials)
(“myofibroblastic sarcoma” OR “myofibroblastic sarcomas” OR myofibrosarcoma*):ti,ab,kw OR MeSH descriptor: [Fibrosarcoma] explode all trees 18 hits
Search strategy Web of Science
TS = (“myofibroblastic sarcoma” OR “myofibroblastic sarcomas” OR myofibrosarcoma*) 404 hits

Tiab, title/abstract (pubmed); tw, textwords (pubmed); mh, MeSH Terms (pubmed); TS, Topic (Web of Science); Exp, Explode (Embase); kw, keywords (Central).

**Table 2:** Eligibility criteria.

**P**atients	Histology of LGMS in the extremities, excluding tumors of the trunk
**I**ntervention	Surgical treatment
**C**omparison	Wide resection vs. marginal resection of the tumor
**O**utcome	Local recurrence, metastasis, evidence of disease
**S**tudy design	Randomized controlled trials, prospective trials, retrospective studies, case reports, systematic reviews

**Table 3:** Patient characteristics, treatment and outcome of cases of LGMS of the extremities located in the soft tissue.

Authors	Study Design	Year	Case no.	Gender	Age	Site of Tumor	Size (cm)	Treatment	Local Recurrence	Metastases	Outcome	FU (Months)
(a) Patient characteristics, treatment and outcome of cases of LGMS of the extremities located in the soft tissue												
Mentzel et al. [3]	RS	1998	1	F	29	Right supraclavicular	8	LE	(−)	(−)	NSR	20
			2	M	31	Left thigh	4	LE	(−)	(−)	NSR	22
			3	M	52	Left shoulder	4	LE	(−)	(−)	NA	NA
			4	M	50	Right arm	3	LE + RT	(−)	(−)	NA	NA
			5	F	33	Left inner thigh	3.2	LE + RT	(−)	(−)	NSR	12
Montgomery et al. [16]	RS	2001	6	M	42	Axilla subcutis	5	LE	(−)	(−)	NSR	36
			7	M	69	Leg	NA	WR + CT	(+)	(−)	LR at 8 months, then RT, further LR at 70 months, then amp	70
			8	M	57	Anterior thigh	4	WR + RT	(−)	(−)	NSR	144
			9	M	64	Arm	1.5	LE	(−)	(−)	NSR	48
Meng et al. [22]	RS	2007	10	F	30	Shoulder	3.7	NA (SP)	(−)	(−)	NSR	22
			11	M	9	Scapular area	3	NA (SP)	(−)	(−)	NSR	41
			12	F	40	Groin	3	NA (SP) + CT	(−)	(−)	NSR	34
Nagata et al. [23]	CR	2008	13	M	36	Palm (ST)	2.5 × 1.5	LE	(−)	(−)	NSR	25
Nakashima et al. [24]	CR	2012	14	F	43	Parapatellar tendon	3	WR + RT	(−)	(−)	NSR	36
Oylumlu et al. [8]	CR	2014	15	M	36	Inguinal region	NA	NA (SP)	(+)	(+)	LR and death	NA
Wechalekar et al. [25]	CR	2014	16	M	62	Multicentric (knees, shoulder, hips, sartorius muscle)	NA	LE (ME)	(−)	(−)	NA	NA
Cai et al. [26]	RS	2016	17	M	6	Left hip	5	WR	(−)	(−)	NSR	11
Wang et al. [9]	RS	2019	18	NA		Right shoulder	8.9 × 6.8	NA (SP)	(+)	(−)	LR	48
			19	NA		Right thigh	5.6 × 7.4	NA (SP)	(+)	(−)	IMT translate into LGMS after LR for 3 times, 8 LR within 8 years	192
Yonezawa et al. [10]	CR	2020	20	F	69	Left M. levator scapulae	2.5 × 3.5	WR	(−)	(−)	NSR	NA
Kim et al. [27]	RS	2021	21	F	57	Lt. medial elbow	2.9 × 2.4 × 2.0	WR	(−)	(−)	NSR	NA
			22	M	68	Rt. Deltoid	2.9 × 1.2 × 1.1	WR	(−)	(−)	NSR	18
			23	M	50	Lt. shoulder	0.7 × 0.5 × 0.4	WR	(−)	(−)	NSR	25
			24	F	45	Rt. forearm	NA	WR	(−)	(−)	NSR	18
			25	F	20	Lt. shoulder	2.8 × 2.4 × 2.0	WR	(−)	(−)	NSR	24
			26	M	68	Rt. shoulder	2.3 × 2.1 × 1.2	WR	(−)	(−)	NSR	60
			27	F	36	Lt. lateral thigh	1.7 × 1.4 × 0.8	WR	(−)	(−)	NSR	15
			28	M	62	Rt. Inguinal	5.9 × 5.8 × 4.9	WR	(−)	(−)	NSR	10
			29	M	61	Rt. Forearm	3.7 × 3.3 × 2.8	WR	(−)	(−)	NSR	38
			30	M	58	Lt. hand 2nd webspace	3.9 × 3.7 × 2.6	LE (En bloc excision)	(−)	(−)	NSR	18
			31	F	70	Distal thigh	2.3 × 2.0, 0.5	WR	(−)	(−)	NSR	15
			32	F	46	Rt. Forearm	1.2 × 0.5 × 1.1	WR	(−)	(−)	NSR	20
Present case	CR	2020	33	M	28	Thumb	2 × 1.5	WR + PS	(+)	(−)	NSR	38
Kito et al. [28]	RS, MCS	2023	34	F	30	Ankle	1.6	LE (ME)	(−)	(−)	NSR	83
			35	M	11	Upper Arm	2.8	WR	(−)	(−)	NSR	96
			36	F	19	Thigh	3.7	WR	(−)	(−)	NSR	94
			37	M	12	Foot	2.8	WR	(−)	(−)	NSR	118
			38	F	79	Forearm	4	WR + RT (48 Gy)	(−)	(−)	NSR	50
			39	F	27	Upper Arm (Relapse at initial presentation)	2.5	WR	(−)	(−)	NSR	32
			40	F	19	Groin	3	WR	(−)	(−)	NSR	55
			41	F	74	Lower leg	9.5	WR + RT (60 Gy)	(+)	(−)	AED	54
			42	M	33	Thigh	2.8	WR	(−)	(−)	NSR	73
			43	F	26	Axilla	7	LE (ME)	(+)	(−)	NSR	181
			44	F	86	Thigh	10	WR + RT (66 Gy)	(−)	(−)	NSR	88
(b) Patient characteristics, treatment and outcome of cases of LGMS of the extremities with local tissue invasion of the bone.												
Montgomery et al. [16]	RS	2001	45	M	65	Tibia (B)	11	LE	(+)	(−)	LR at 24 months, then amp, then NSR 172 at months	172
Watanabe et al. [29]	RS	2001	46	M	60	Distal Femur (B)	5	WR + CT	(−)	(−)	NSR	NA
			47	F	63	Distal Femur (B)	9	LE	(−)	(−)	NSR	NA
San Miguel et al. [30]	CR	2004	48	F	51	Distal Phalanx	1.8	Amp	(−)	(−)	NSR	28
Meng et al. [22]	RS	2007	49	M	14	Femur	10	NA (SP) + CT	(+)	(−)	LR	20
			50	F	30	Femur	4	NA (SP) + CT	(+)	(−)	LR 2x	29
Arora et al. [31]	CR	2010	51	F	38	Femur (B)	20 × 10	WR + prosthesis	(−)	(−)	AED	NA
Saito et al. [32]	CR	2013	52	F	50	Distal femur (B)	NA	WR + prosthesis	(−)	(−)	NSR	15
Cai et al. [26]	RS	2016	53	F	43	Right tibia (B)	4	LE (ILR + bone graft)	(+)	(−)	LR	33
Wang et al. [9]	RS	2019	54	NA		Right scapula (B)	13 × 13.5	NA (SP)	(−)	(+)	Pulmonary metastasis before operation	NA
			55	NA		Left distal femur (B)	NA	NA (SP)	(−)	(+)	Pulmonary metastasis after 8 months of operation	NA
			56	NA		Right distal femur (B)	NA	NA (SP)	(−)	(+)	Pulmonary metastasis after 56 months of operation	56
			57	NA		Left distal femur (B)	NA	NA (SP)	(+)	(+)	LR and bone metastasis at 17 months	17
Kim et al. [27]	RS	2021	58	M	27	Rt.3rd finger proximal phalanx (B)	2.2 × 2.0 × 1.0	LE (ME, En bloc excision limb salvage surgery)	(+)	(−)	LR	71
Gao et al. [7]	CR	2022	59	F	30	Femoral head neck junction (B)		LE (ILE, Hip arthroscopy)	(−)	(−)	NSR	6

CR, case report; M, male; F, female; B, bone; ST, soft tissue; WR, wide resection; NA, not applicable; CT, chemotherapy; LE, local excision; ME, marginal excision; LR, local recurrence; Amp, amputation; RT, radiotherapy; NSR, no sign of recurrence; DOD, dead of disease; AED, alive with evidence of disease; IMT, inflammatory myofibroblastic tumor; MCS, multicenter study; RS, retrospective study; SP, surgical procedure; ST, soft tissue; PS, plastic surgery; (−), no; (+), yes.

**Table 4:** Critical appraisal of cases according to Moola et al. [33].

Author	Case No.	Study Design	Were the Patient’s Demographic Characteristics Clearly Described?	Was the Patient’s History Clearly Described and Presented as a Timeline?	Was the Current Clinical Condition of the Patient on Presentation Clearly Described?	Were Diagnostic Tests or Assessment Methods and the Results Clearly Described?	Was the Intervention(s) or Treatment Procedure(s) Clearly Described?	Was the Post-Intervention Clinical Condition Clearly Described?	Were Adverse Events (Harms) or Unanticipated Events Identified and Described?	Does the Case Report Provide Takeaway Lessons?	Overall Appraisal: Include Exclude Seek further Info
Mentzel et al. [3]	1	RS	Yes	Yes	No	Yes	Yes	Yes	No	Yes	Include
	2		Yes	Yes	No	Yes	Yes	Yes	No	Yes	Include
	3		Yes	Yes	No	Yes	Yes	No	No	Yes	Include
	4		Yes	Yes	No	Yes	Yes	No	No	Yes	Include
	5		Yes	Yes	No	Yes	Yes	Yes	No	Yes	Include
Montgomery et al. [16]	6	RS	Yes	No	Yes	Yes	Yes	Yes	Yes	Yes	Include
	7		Yes	No	Yes	Yes	Yes	Yes	Yes	Yes	Include
	8		Yes	No	Yes	Yes	Yes	Yes	Yes	Yes	Include
	9		Yes	No	Yes	Yes	Yes	Yes	Yes	Yes	Include
	10		Yes	No	Yes	Yes	Yes	Yes	Yes	Yes	Include
Watanabe et al. [29]	11	RS	Yes	No	No	Yes	Yes	Yes	No	Yes	Include
	12		Yes	No	No	Yes	Yes	Yes	No	Yes	Include
San Miguel et al. [30]	13	CR	Yes	Yes	Yes	Yes	Yes	Yes	Yes	Yes	Include
Meng et al. [22]	14	RS	Yes	No	No	Yes	Yes	Yes	No	Yes	Include
	15		Yes	No	No	Yes	Yes	Yes	No	Yes	Include
	16		Yes	No	No	Yes	Yes	Yes	No	Yes	Include
	17		Yes	No	No	Yes	Yes	Yes	No	Yes	Include
	18		Yes	No	No	Yes	Yes	Yes	No	Yes	Include
Nagata et al. [23]	19	CR	Yes	Yes	Yes	Yes	No	No	No	Yes	Include
Arora et al. [31]	20	CR	Yes	Yes	Yes	Yes	No	No	No	Yes	Include
Nakashima et al. [24]	21	CR	Yes	Yes	Yes	Yes	Yes	Yes	Yes	Yes	Include
Saito et al. [32]	22	CR	Yes	Yes	Yes	Yes	Yes	No	Yes	Yes	Include
Oylumlu et al. [8]	23	CR	Yes	Yes	Yes	Yes	No	No	Yes	Yes	Include
Wechalekar et al. [25]	24	CR	Yes	Yes	Yes	Yes	No	No	No	Yes	Include
Cai et al. [26]	25	RS	Yes	No	No	Yes	Yes	Yes	No	Yes	Include
	26		Yes	No	No	Yes	Yes	Yes	No	Yes	Include
Wang et al. [9]	27	RS	No	No	No	Yes	No	No	Yes	Yes	Include
	28		No	No	No	Yes	No	No	Yes	Yes	Include
	29		No	No	No	Yes	No	Yes	Yes	Yes	Include
	30		No	No	No	Yes	No	Yes	Yes	Yes	Include
	31		No	No	No	Yes	No	Yes	Yes	Yes	Include
	32		No	No	No	Yes	No	Yes	Yes	Yes	Include
Yonezawa et al. [10]	33	CR	Yes	Yes	Yes	Yes	Yes	Yes	Yes	Yes	Include
Kim et al. [27]	34	RS	Yes	No	No	Yes	Yes	Yes	No	Yes	Include
	35		Yes	No	No	Yes	Yes	Yes	No	Yes	Include
	36		Yes	No	No	Yes	Yes	Yes	No	Yes	Include
	37		Yes	No	No	Yes	Yes	Yes	No	Yes	Include
	38		Yes	No	No	Yes	Yes	Yes	No	Yes	Include
	39		Yes	No	No	Yes	Yes	Yes	No	Yes	Include
	40		Yes	No	No	Yes	Yes	Yes	No	Yes	Include
	41		Yes	No	No	Yes	Yes	Yes	No	Yes	Include
	42		Yes	No	No	Yes	Yes	Yes	No	Yes	Include
	43		Yes	No	No	Yes	Yes	Yes	No	Yes	Include
	44		Yes	No	No	Yes	Yes	Yes	No	Yes	Include
	45		Yes	No	No	Yes	Yes	Yes	No	Yes	Include
	46		Yes	No	No	Yes	Yes	Yes	No	Yes	Include
Present case	47	CR	Yes	Yes	Yes	Yes	Yes	Yes	Yes	Yes	Include
Gao et al. [7]	48	CR	Yes	Yes	Yes	Yes	Yes	Yes	Yes	No	Include
Kito et al. [28]	49	RS, MC	Yes	No	No	Yes	Yes	Yes	No	Yes	Include
	50		Yes	No	No	Yes	Yes	Yes	No	Yes	Include
	51		Yes	No	No	Yes	Yes	Yes	No	Yes	Include
	52		Yes	No	No	Yes	Yes	Yes	No	Yes	Include
	53		Yes	No	No	Yes	Yes	Yes	No	Yes	Include
	54		Yes	No	No	Yes	Yes	Yes	No	Yes	Include
	55		Yes	No	No	Yes	Yes	Yes	No	Yes	Include
	56		Yes	No	No	Yes	Yes	Yes	No	Yes	Include
	57		Yes	No	No	Yes	Yes	Yes	No	Yes	Include
	58		Yes	No	No	Yes	Yes	Yes	No	Yes	Include
	59		Yes	No	No	Yes	Yes	Yes	No	Yes	Include

**Table 5:** Summary of the immunohistochemical stainings and their results, performed at the Department of Pathology, University Hospital Münster (using the primary tumor material). [+++, strong positivity; ++, moderate positivity; +, weak positivity; −, negative immunohistochemical reaction].

Marker	Results of Immunhistochemistry
Smooth muscle actin	+++
Desmin	+++
CD10	+++
CD34	+ (10% of tumor cells)
D2-40	++
ERG	+++
INI-1 (nuclear)	+++
TFE3	+ (focal)
p53	wild type
Ki-67	15–20%
ALK1	−
Caldesmon	−
Calponin	−
ß-Catenin	−
CD31	−
EMA	−
FosB	−
H3K27me3	−
HHV8	−
Muc-4	unspecific
S-100	−
Stat-6	−

## Data Availability

No new data were created or analyzed in this study. Data sharing is not applicable to this article.
